# Central Inflammation and Leptin Resistance Are Attenuated by Ginsenoside Rb1 Treatment in Obese Mice Fed a High-Fat Diet

**DOI:** 10.1371/journal.pone.0092618

**Published:** 2014-03-27

**Authors:** Yizhen Wu, Yinghua Yu, Alexander Szabo, Mei Han, Xu-Feng Huang

**Affiliations:** 1 Illawarra Health and Medical Research Institute, School of Medicine, University of Wollongong, Wollongong, New South Wales, Australia; 2 Schizophrenia Research Institute, Sydney, New South Wales, Australia; 3 ANSTO LifeSciences, Australian Nuclear Science and Technology Organisation, Sydney, New South Wales, Australia; University of Cordoba, Spain

## Abstract

A low-grade pro-inflammatory state is at the pathogenic core of obesity and type 2 diabetes. We tested the hypothesis that the plant terpenoid compound ginsenoside Rb1 (Rb1), known to exert anti-inflammatory effects, would ameliorate obesity, obesity-associated inflammation and glucose intolerance in the high-fat diet-induced obese mouse model. Furthermore, we examined the effect of Rb1 treatment on central leptin sensitivity and the leptin signaling pathway in the hypothalamus. We found that intraperitoneal injections of Rb1 (14 mg/kg, daily) for 21 days significantly reduced body weight gain, fat mass accumulation, and improved glucose tolerance in obese mice on a HF diet compared to vehicle treatment. Importantly, Rb1 treatment also reduced levels of pro-inflammatory cytokines (TNF-α, IL-6 and/or IL-1β) and NF-κB pathway molecules (p-IKK and p-IκBα) in adipose tissue and liver. In the hypothalamus, Rb1 treatment decreased the expression of inflammatory markers (IL-6, IL-1β and p-IKK) and negative regulators of leptin signaling (SOCS3 and PTP1B). Furthermore, Rb1 treatment also restored the anorexic effect of leptin in high-fat fed mice as well as leptin pSTAT3 signaling in the hypothalamus. Ginsenoside Rb1 has potential for use as an anti-obesity therapeutic agent that modulates obesity-induced inflammation and improves central leptin sensitivity in HF diet-induced obesity.

## Introduction

Obesity has reached epidemic proportions and is an important risk factor for the development of type 2 diabetes, cardiovascular disease and cancer. It is generally accepted that a low-grade pro-inflammatory state is at the pathogenic core of obesity and type 2 diabetes [1], [2]. This inflammatory response includes elevated levels of pro-inflammatory cytokines, such as tumor necrosis factor alpha (TNF-α), interleukin 1 beta (IL-1β) and interleukin 6 (IL-6), and activation of the nuclear factor kappa-light-chain-enhancer of activated B cells (NF-κB) signaling pathway, including inhibitor kappa B alpha (IκBα) and IκB kinase (IKK) [3], [4]. The activation of pro-inflammatory cytokines and NF-κB signaling pathway mediate the transcription of the suppressor of cytokine signaling 3 (SOCS3) and protein-tyrosine phosphatase 1B (PTP1B), negative regulators of insulin and leptin signaling, which induce insulin and leptin resistance in peripheral tissues and the central nervous system [5], [6], [7]. Obesity associated inflammation in white adipose tissue and the liver leads to glucose intolerance, insulin resistance and metabolic dysfunction [2], [8], [9]. Over-nutrition and obesity also leads to hypothalamic inflammation and stimulation of local pro-inflammatory NF-κB signaling, resulting in the dysfunction of hypothalamic neurons [4], [5]. Furthermore, recent studies have shown that induction of inflammation in the hypothalamus results in experimental obesity, resistance to the anorexigenic hormone leptin, peripheral insulin resistance and defective regulation of food intake and energy expenditure [10], [11], [12].

Targeted deletion of certain genes important for mediating inflammatory responses protect against the development of hyperglycemia, insulin resistance and obesity in obese mouse models. Disruption of the gene encoding IKK and the innate immune system receptor Toll-like receptor (TLR)-4 in mice confers protection from insulin and leptin resistance, and obesity in mouse models [5], [13]. Also, inhibition of NF-κB signaling using high-dose salicylates confers protection from obesity-induced inflammation and insulin resistance in mice [14]. Activation of hypothalamic NF-κB by central injection of a constitutively active IKKβ lentiviral vector interrupts central leptin and insulin signaling, while genetic or viral vector mediated suppression of IKK within the mediobasal hypothalamus protects against obesity and glucose intolerance in mice [5]. Therefore, compounds that attenuate the peripheral and hypothalamic inflammation associated with obesity may prove useful in the management of patients with obesity and type 2 diabetes.

Some plant-derived triterpenoids are anti-inflammatory and inhibit the NF-κB signaling pathway [15]. The tetracyclic triterpenoid ginsenoside Rb1 (Rb1) is the major bioactive compound extracted from ginseng [16], [17]. This compound inhibits inflammation in *in vitro* and *in vivo* models, including anti-inflammatory effects on aortic smooth muscle exposed to TNF-α [18], the colon of colitis mice [19], and brain tissue in an cerebral ischemia animal model [20]. In high-fat diet-induced obese rats, Rb1 significantly reduces food intake and body weight gain [21]. A study by Lin [22] shows that in high-fat diet-induced obese mice, Rb1 significantly reduces weight gain, blood glucose and total cholesterol. However, it is unknown whether Rb1 can improve obesity-associated inflammation and central leptin resistance.

## Materials and Methods

### Animal care and treatment

C57Bl/6 male mice (6 weeks old, average body weight of 19.6±1.4 g) were obtained from the Animal Resources Centre (Perth, Western Australia) and housed in environmentally controlled conditions (temperature 22°C, 12 hour light/dark cycle). All animals were fed a lab chow (LC) diet (5% fat, Vella Stock Feeds, Doonside, NSW, Australia) ad libitum for one week and then fed a high-fat (HF) for 16 weeks (The HF diet contained 40% of energy as fat, with the fat content consisting of half lard and half sunflower oil. The proportion of saturated fat, n-6 polyunsaturated fat, n-3 polyunsaturated fat and monounsaturated fat were 12%, 16%, 0.4% and 11% respectively. SF11-095, Specialty Feeds, Western Australia). After 16 weeks of HF diet, obese mice (average body weight of 44.18±2.60 g) were randomized into two groups (n = 16 per group) and treated with either daily intraperitoneal (ip) injections of Rb1 (14 mg/kg, based on a Rb1 dose (10 mg/kg) described previously in rats [21], and using a body surface area ratio of 0.14 from rat to mouse [23]) or vehicle (saline) for 21 days. This study also included a parallel control group of age-matched mice fed a LC diet. Rb1 purified by high-performance liquid chromatography (HPLC) to ≥98% was purchased from Jilin University in China. During Rb1 treatment the animal's food intake and body weight were recorded daily. All procedures were approved by the Animal Ethics Committee of the University of Wollongong, NSW, Australia, and complied with the *Australian Code of Practice for the Care and Use of Animals for Scientific Purposes*. The approval ID for this study is AE10/08.

### Glucose tolerance test (GTT)

On day 18 of Rb1 treatment, the mice were injected intraperitoneally with glucose at a dose of 0.5 g/kg after an overnight fast. Blood samples were taken from the tail vein, and blood glucose concentration determined using a glucometer (Freestyle; Abbott Diabetes Care, Alameda, CA) at 0 (fasting), 30, 60 and 120 minutes after glucose injection.

### Central leptin sensitivity

Central leptin sensitivity was examined in moderate and severely obese mice. For moderate obesity, mice were fed a HF diet for 8 weeks followed by an acute treatment of Rb1 (14 mg/kg/day, ip) for 2 days. For severe obesity, the mice were fed a HF diet for 16 weeks and then administered Rb1 (14 mg/kg/day, ip) for 21 days. The central leptin sensitivity test was performed as follows. Mice were anesthetized by isoflurane inhalation and placed in a stereotactic device. An intracerebroventricular (icv) cannula was implanted into the right lateral brain ventricle (0.25 mm posterior and 1.0 mm lateral relative to Bregma and 2.5 mm below the surface of the skull) [24]. Five days after implantation the mice were fasted for 6 hours, and either leptin (0.1 μg/3 μl) or saline (3 μl) was injected into the lateral ventricle through the cannula. Food intake and body weight were measured for 24 hours after the leptin or vehicle injection.

### Blood and Tissue collection

Following a further four day interval after examining central leptin sensitivity, mice were fasted for 6 hours, administered an icv injection of either leptin (0.1 μg/3 μl) or saline (3 μl) and then euthanized 1 hour later for tissue collection. Blood, white adipose tissue, liver and brain tissue were collected. The plasma from mice receiving saline icv injections was collected after centrifugation at 3000rpm for 15 minutes. Plasma and other tissues were stored at −80°C for further analyses.

Using a standard mouse brain atlas [24], 500 μm frozen brain sections were cut from Bregma -1.22 mm to -2.72 mm using a cryostat at a temperature of −18°C. The mediobasal hypothalamus was dissected and then collected using a Stoelting Brain Punch (#57401, 0.5 mm diameter, Wood Dale, Stoelting Co, USA) in an overlapping pattern over the 3rd ventricle [25].

### Determination of plasma leptin, insulin, peptide YY (PYY) and adiponectin

Plasma leptin, insulin and PYY were measured using the mouse metabolic magnetic bead panel kit (Merck Millipore, MA), and adiponectin was assayed with the mouse single plex adiponectin kit (Merck Millipore).

### Histological analysis and morphometry

Epididymal fat was fixed in 10% buffered formaldehyde and then embedded in paraffin. Tissue sections (5 μm) were cut and mounted onto polysine slides. The sections were stained with hematoxylin and eosin and photographed at 100× magnification. Using the image analysis software Image J 1.46r (http://rsbweb.nih.gov/ij/download.html), two fields per section and six sections per fat mass were analyzed to quantify the area and number of adipocytes.

### Western blot analysis

As described in our previous study [26], tissue protein was extracted using NP-40 Lysis Buffer. The following antibodies were used: TNF-α (sc-8301), IL-1β (sc-7884), and IL-6 (sc-7920) from Santa Cruz Biotechnology (Dallas, TX); and p-IκBα (#2859), p-IKK (#2697), p-STAT3 (#9145), SOCS3 (#2932), and p-FOXO1 (#9461) from Cell Signaling Technology (Beverly, MA). Bands corresponding to the proteins of interest were analyzed using the automatic imaging analysis system Quantity One (Bio-Rad Laboratories, Hercules, CA). All quantitative analyses were normalized to β-actin as described in our previous study [26].

### Quantitative real-time PCR (qPCR)

Total mediobasal hypothalamic RNA was extracted using the Aurum total RNA mini kit (Bio-Rad Laboratories, Hercules, CA) and reverse-transcribed to first-strand complementary DNA with the high-capacity cDNA reverse transcription kit (AB Applied Biosystems, Carlsbad, CA), according to the manufacturer's instructions. qPCR was performed in a 20 μl final reaction volume using SYBR green I master in a Lightcycler 480 (F. Hoffmann-La Roche Ltd, Basel, Switzerland). Primers used are listed in Table S1. Amplification was carried out with 45 cycles of 95°C for 10 seconds, 60°C for 30 seconds and 72°C for 30 seconds. The mRNA expression levels for hypothalamic neuropeptides were normalized to gamma actin, which served as the internal control. Experiments were performed in triplicate. The level of expression for each gene was calculated using the comparative threshold cycle value (Ct) method, using the formula 2^−ΔΔCt^ as described previously [27], [28].

### Statistical analysis

Data were analyzed using the SPSS 19 statistical package (SPSS, Chicago, IL). The two-tailed student's t-test was used to compare food intake, adipose tissue histology and weight, inflammatory markers in epididymal adipose tissue and liver, and hypothalamic neuropeptides. One-way analysis of variance (ANOVA) was followed by the post hoc Tukey–Kramer honestly significant difference (HSD) test was used to analyze final body weight gain, plasma cytokines, central inflammatory markers, and central leptin sensitivity. A *p*<0.05 was regarded as statistically significant, and *p*<0.10 were considered a trend. Values are expressed as mean ± SEM.

## Results

### Rb1 treatment lowered food intake and prevented weight gain and fat deposition in obese mice on a HF diet

**Figure 1:**
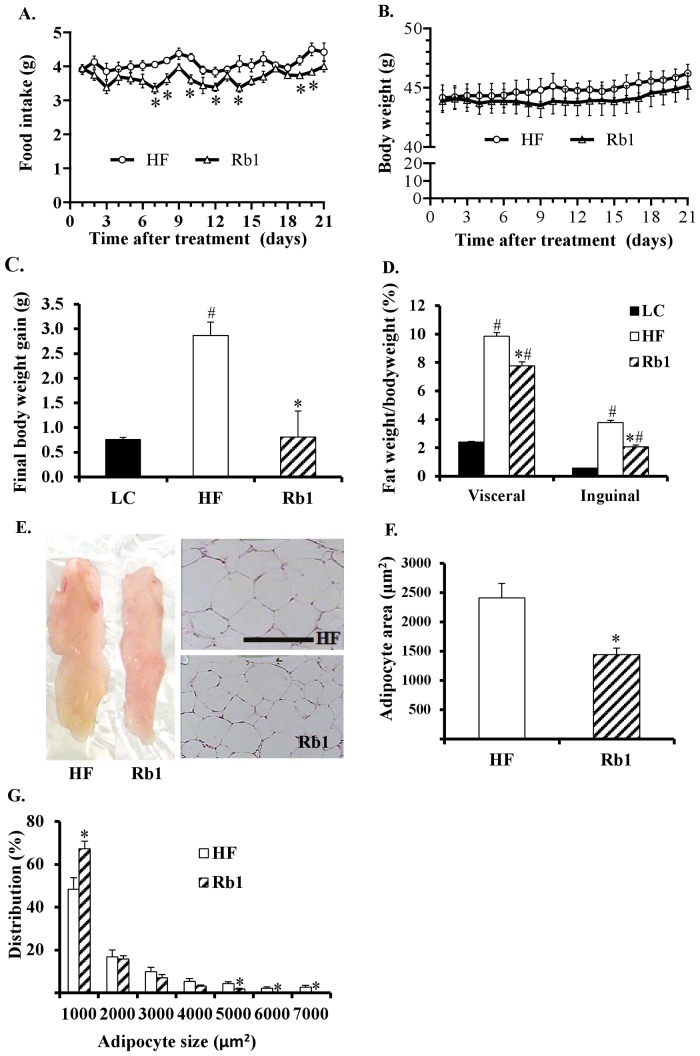
Effect of chronic administration of Rb1 on food intake, body weight and fat in obese mice fed a HF diet for 16 weeks. Rb1 treatment reduced food intake (A), final body weight gain (C), and visceral and inguinal fat mass (D) (n = 8). Panel B: Body weight of obese mice with or without Rb1 chronic treatment for 21 days. Panel E: Photographs of epididymal fat tissue, and hematoxylin and eosin staining of epididymal fat tissue, scale bar: 100 μm. Panel F: Adipocyte area in epididymal fat. Panel G: Frequency distribution of adipocyte surface area in epididymal fat. *p<0.05 vs. HF control group, #p<0.05 vs. LC lean control.

Overall, Rb1 treatment reduced average food intake by 11% (*p*<0.05) in HF diet fed mice, and a reduction of food intake was observed on days 7, 8, 10, 12, 14, 19 and 20 of Rb1 treatment (all *p*<0.05, Fig. 1A) compared with HF control group. Rb1 treatment significantly reduced body weight gain (Fig. 1C) and visceral and subcutaneous (inguinal) fat deposition (Fig. 1D and Table 1) in mice maintained on a HF diet. Rb1 treatment also decreased the size of adipocytes (an indication of fat storage), with adipocytes from epididymal visceral fat pads being significantly smaller in response to Rb1 treatment (Fig. 1E and F). The distribution of adipocytes by cell surface area showed a higher proportion of small-sized cells (1,000 μm^2^) and a lower proportion of larger-sized cells (5,000–7,000 μm^2^) in the Rb1-treated group compared to the HF group (Fig. 1 G).

**Table 1:** Weight of fat pads in HF diet-induced obese mice with and without ginsenoside Rb1 treatment.

	HF	Rb1	*p*-value
Visceral fat (g)	3.78±0.17	2.79±0.12^*^	<0.001
Epididymal fat (g)	2.10±0.06	1.50±0.08^*^	<0.001
Perirenal fat (g)	0.85±0.06	0.56±0.04^*^	<0.001
Mesenteric fat (g)	0.83±0.06	0.73±0.06	0.236
Inguinal fat (g)	1.50±0.08	0.80±0.05^*^	<0.001

HF: high-fat diet–induced obese mice; Rb1: high-fat diet-induced obese mice treated with ginsenoside Rb1. Visceral fat includes epididymal, perirenal and mesenteric. *p<0.05 vs. HF group. Data are presented as Mean±SEM.

### Rb1 treatment improved blood hormone profiles for energy balance regulation

**Figure 2:**
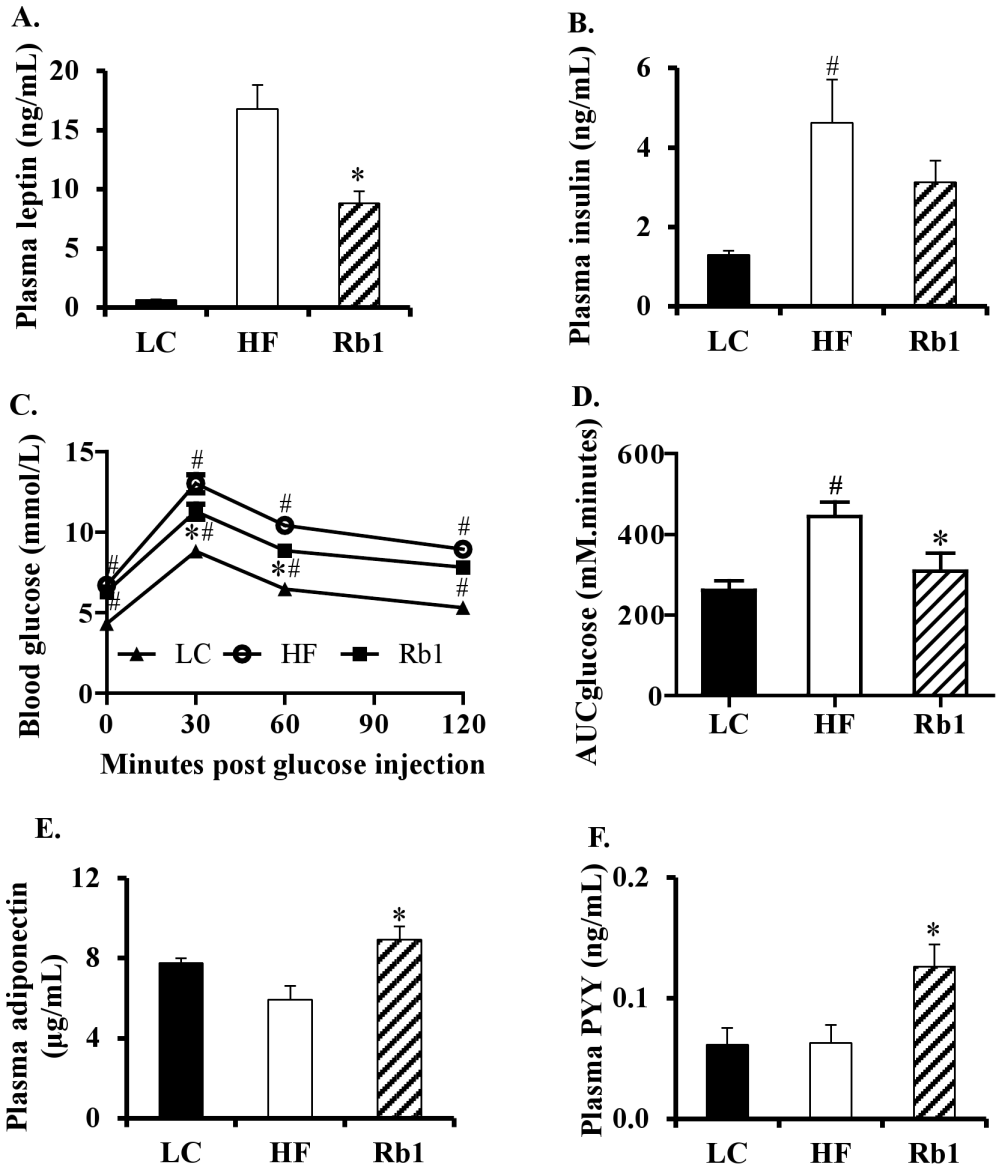
Chronic administration of Rb1 improved plasma profiles and glucose tolerance in obese mice fed a HF diet for 16 weeks. Rb1 decreased plasma leptin (A) and insulin (B), improved glucose tolerance (C) and AUC (D), and increased adiponectin (E) and PYY (F) in obese mice (n = 8) fed a HF diet for 16 weeks. *p<0.05 vs. HF control group; ^#^ *p*<0.05 vs. LC diet control group. Data are presented as mean ± SEM. Area under the curve for glucose (AUCglucose) was calculated using the trapezoidal rule.

HF diet-induced hyperleptinemia was significantly decreased by Rb1 treatment (Fig. 2A). Plasma insulin was elevated in HF diet-induced obese mice, but Rb1 did not significantly reverse hyperinsulinemia in these animals (Fig. 2B). To evaluate the functional outcome of Rb1 treatment on glucose homeostasis, we conducted a glucose tolerance test (GTT). Blood glucose was reduced by Rb1 treatment at the 30 and 60 minute time points of the GTT (Fig. 2C). The blood glucose area under the curve (AUC) after glucose injection was reduced in Rb1-treated mice compared to HF mice without Rb1 treatment (Fig. 2D). Rb1 also increased plasma adiponectin in HF diet-induced obese mice (Fig. 2E). Circulating concentrations of the anorexigenic peptide PYY were significantly increased in the Rb1 treatment group compared with HF mice (Fig. 2F).

### Rb1 treatment decreased inflammation in adipose tissue and the liver

**Figure 3:**
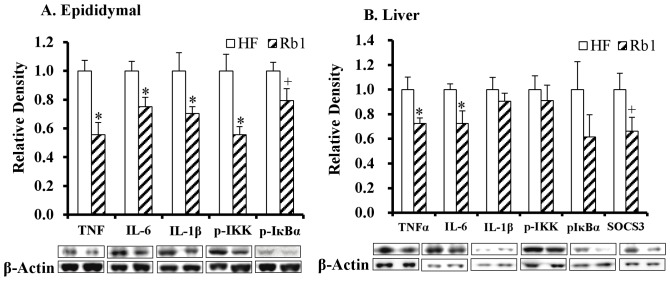
Chronic Rb1 treatment reduced peripheral inflammation in obese mice fed a HF diet for 16 weeks. Protein expression of the pro-inflammatory cytokines TNF-α, IL-6 and IL-1β, as well as the inflammatory signaling molecules p-IKK and p-IκBα in the epididymal fat tissue (A) and liver (B) in HF-induced obese mice with Rb1 chronic treatment (n = 8). *p<0.05 vs. HF diet control group, +*p*<0.10 and >0.05 vs. HF control group. Data are presented as mean ± SEM.

Given the anti-inflammatory properties of Rb1 in aortic smooth muscle, colon and brain [18], [19], [20], we investigated whether Rb1 could reduce low-grade inflammation of adipose and liver tissue in HF diet-induced obese mice. In the epididymal adipose tissue of HF mice treated with Rb1, we found significantly reduced expression of pro-inflammatory cytokines (TNF-α, -44%; IL-6, -25%; IL-1β, −30%; *p*<0.05), as well as the inflammatory signaling molecule p-IKK (−44%; *p*<0.001), compared to HF control mice (Fig. 3A). In a statistical trend, Rb1 treatment lowered p-IκBα expression in epididymal adipose tissue (−21%; *p* = 0.06, Fig. 3A). For the liver of HF mice treated with Rb1, the expression of TNF-α and IL-6 (−28% and -28%; *p*<0.05) was also significantly reduced compared to HF mice without Rb1 treatment (Fig. 3B). Rb1 treatment lowered SOCS3 expression in the liver (−34%; *p* = 0.08, Fig. 3B) compared to HF control mice, in a statistical trend. However, no difference was found in the hepatic expression of IL-1β and p-IKK in HF mice with or without Rb1 treatment (Fig. 3B).

### Rb1 treatment attenuated hypothalamic inflammation and negative regulators of leptin signaling

**Figure 4:**
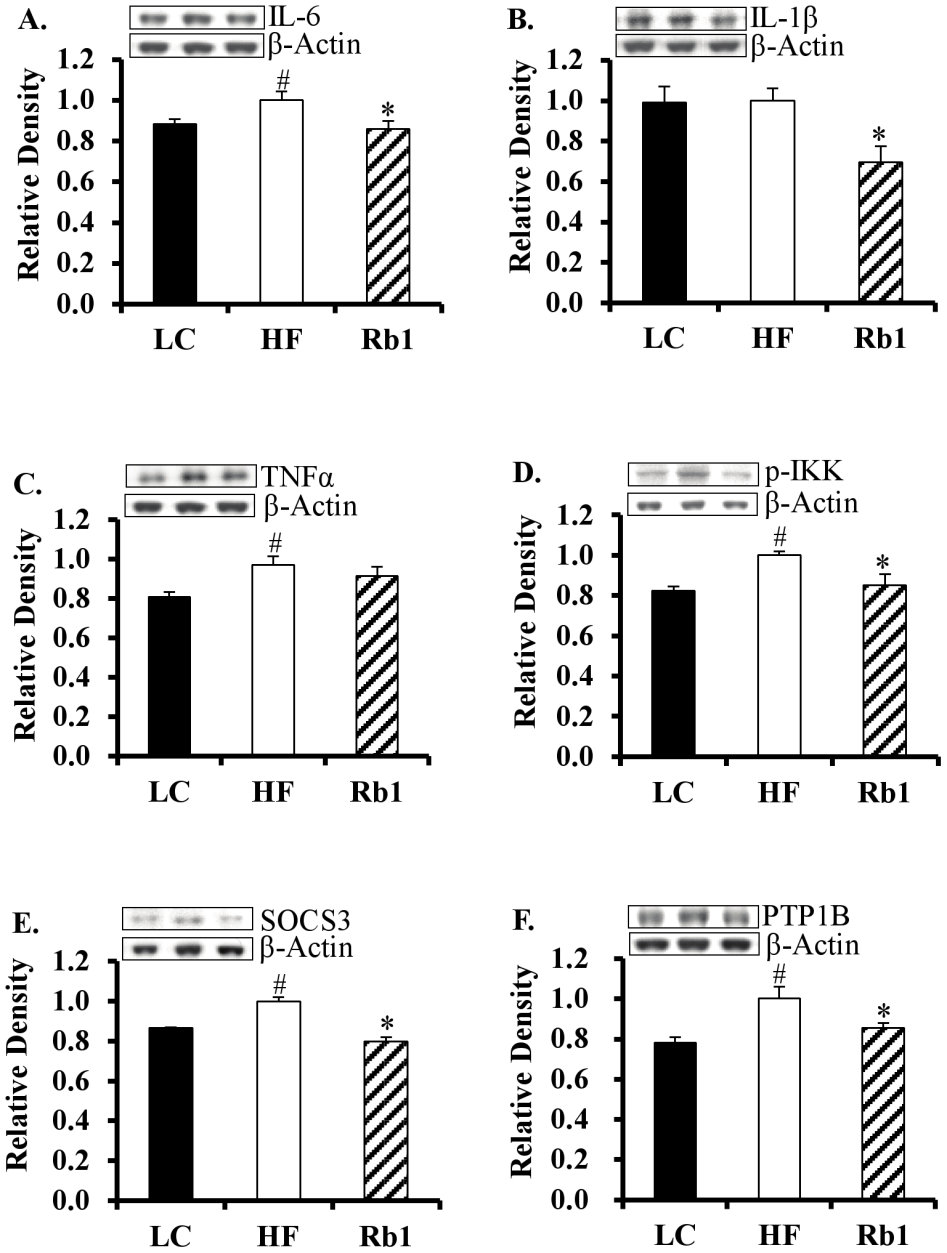
Chronic Rb1 treatment reduced hypothalamic inflammation and negative regulators of leptin signaling in obese mice. Chronic treatment of Rb1 significantly decreased the level of IL-6 (A), IL-1β (B), p-IKK (D), SOCS3 (E) and PTP1B (F) in the mediobasal hypothalamus of obese mice (n = 6–8) fed a HF diet for 16 weeks without leptin icv injection. Panel C: HF diet significantly increased the protein levels of TNFα in the mediobasal hypothalamus of mice. *p<0.05 vs HF group, #*p*<0.05 vs. LC lean control. Data are presented as mean ± SEM.

A HF diet stimulates pro-inflammatory cytokine mRNA expression in the hypothalamus of rodents [4], and here we investigated if Rb1 treatment could attenuate this inflammation. Using western blot analysis, we confirmed that protein levels of IL-6, TNF-α and p-IKK increased in the mediobasal hypothalamus of HF diet-induced obese mice compared with LC diet mice (Fig. 4A, C and D). The protein levels of SOCS3 and PTP1B, negative regulators of leptin signaling, also increased in the mediobasal hypothalamus of HF fed mice (Fig. 4E and F). Importantly, Rb1 treatment significantly decreased the expression of IL-6, IL-1β, p-IKK, SOCS3 and PTP1B (-14%, -31%, -15%, -20% and -14% respectively; *p*<0.05; Fig. 4) in the hypothalamus compared with HF control mice.

### Rb1 treatment improved central leptin sensitivity and leptin signaling

**Figure 5:**
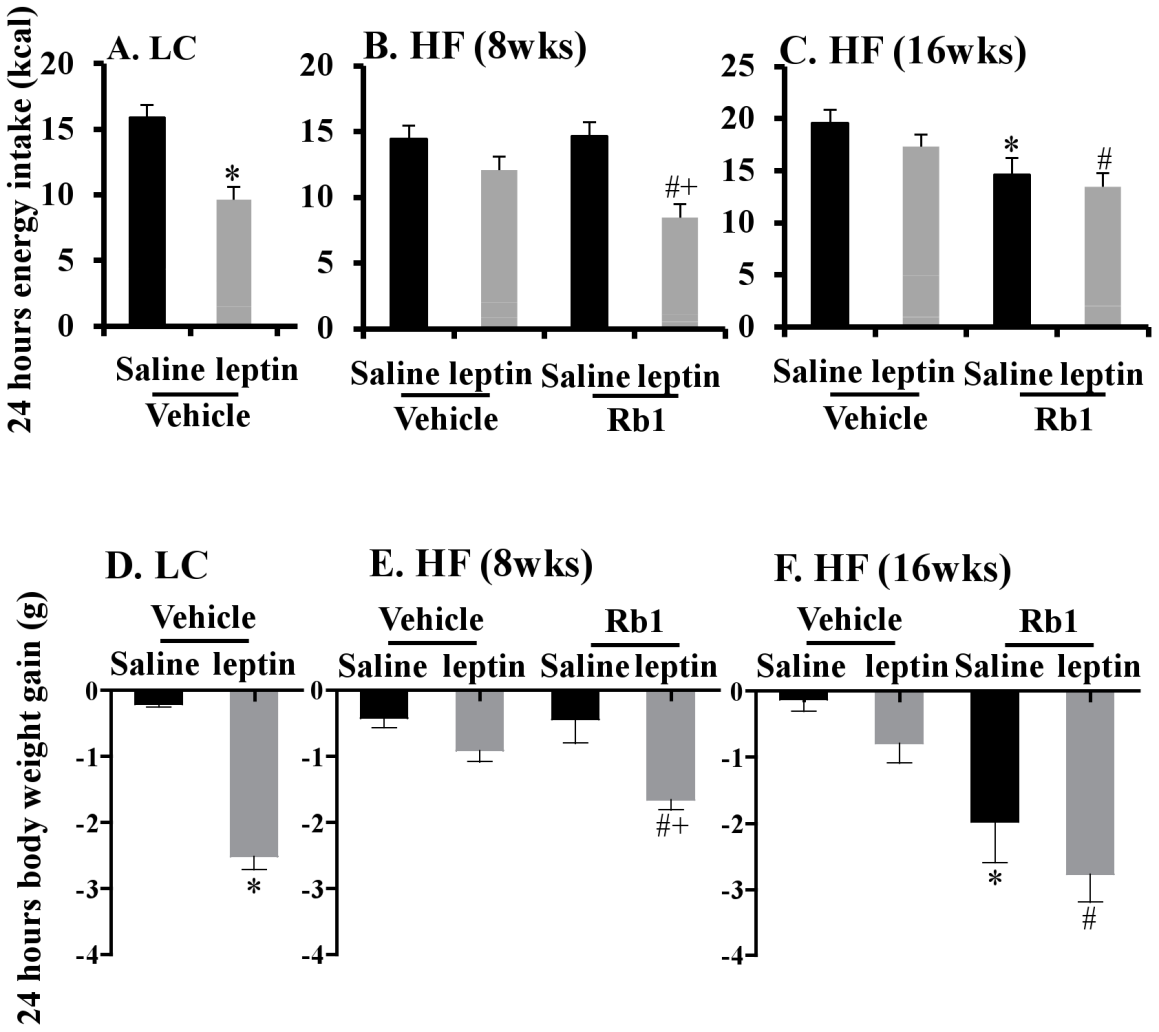
Chronic Rb1 treatment improved central leptin sensitivity in obese mice fed a HF diet for 16 weeks. Energy intake (A–C) and body weight gain (D–F) for 24 hours after the icv injection of leptin or saline in mice fed a LC diet (n = 7–8), in obese mice fed a HF diet for 8 weeks with or without acute treatment of Rb1 (14 mg/kg ip daily for 2 days) (n = 7–8), in obese mice fed a HF diet for 16 weeks with or without chronic treatment of Rb1 (14 mg/kg ip daily for 21 days) (n = 7–8). *p<0.05 vs. (Vehicle + Saline) group; ^#^ *p*<0.05 vs. (Vehicle + leptin) group; and ^+^ *p*<0.05 vs. (Rb1 + Saline) group. Data are presented as mean ± SEM.

To evaluate if Rb1 treatment improved central leptin sensitivity in conjunction with the inhibition of hypothalamic inflammation, central leptin sensitivity was examined at two stages in the development of obesity, at 8 and 16 weeks of HF diet. First, we demonstrated that icv injection of leptin decreased energy intake (-31%; *p*<0.05, Fig. 5A) and body weight gain (*p*<0.05, Fig. 5D) compared with saline injection in lean LC fed mice. Second, after 8 weeks of HF diet leptin did not suppress energy intake and body weight gain in HF control mice (Fig. 5B and E), while acute Rb1 treatment (2 days) restored leptin sensitivity, evidenced by a 41% reduction in energy intake and a very significant reduction in body weight gain following leptin icv injection compared to saline icv injection (*p*<0.05, Fig. 5B and E). Furthermore, acute Rb1 treatment did not significantly suppress overall food intake and body weight (Table S2). In severely obese control mice fed a HF diet for 16 weeks, leptin icv injection did not significantly decrease energy intake and body weight gain compared with saline (*p*>0.05, Fig. 5C and F). With the addition of chronic Rb1 treatment, icv leptin injections significantly decreased energy intake by -22% and decreased body weight gain by -251% compared to leptin injections in obese mice not treated with Rb1 (*p*<0.05, Fig. 5C and F). This suggests that the Rb1 chronic treatment increased the ability of leptin to inhibit energy intake and body weight gain.

**Figure 6:**
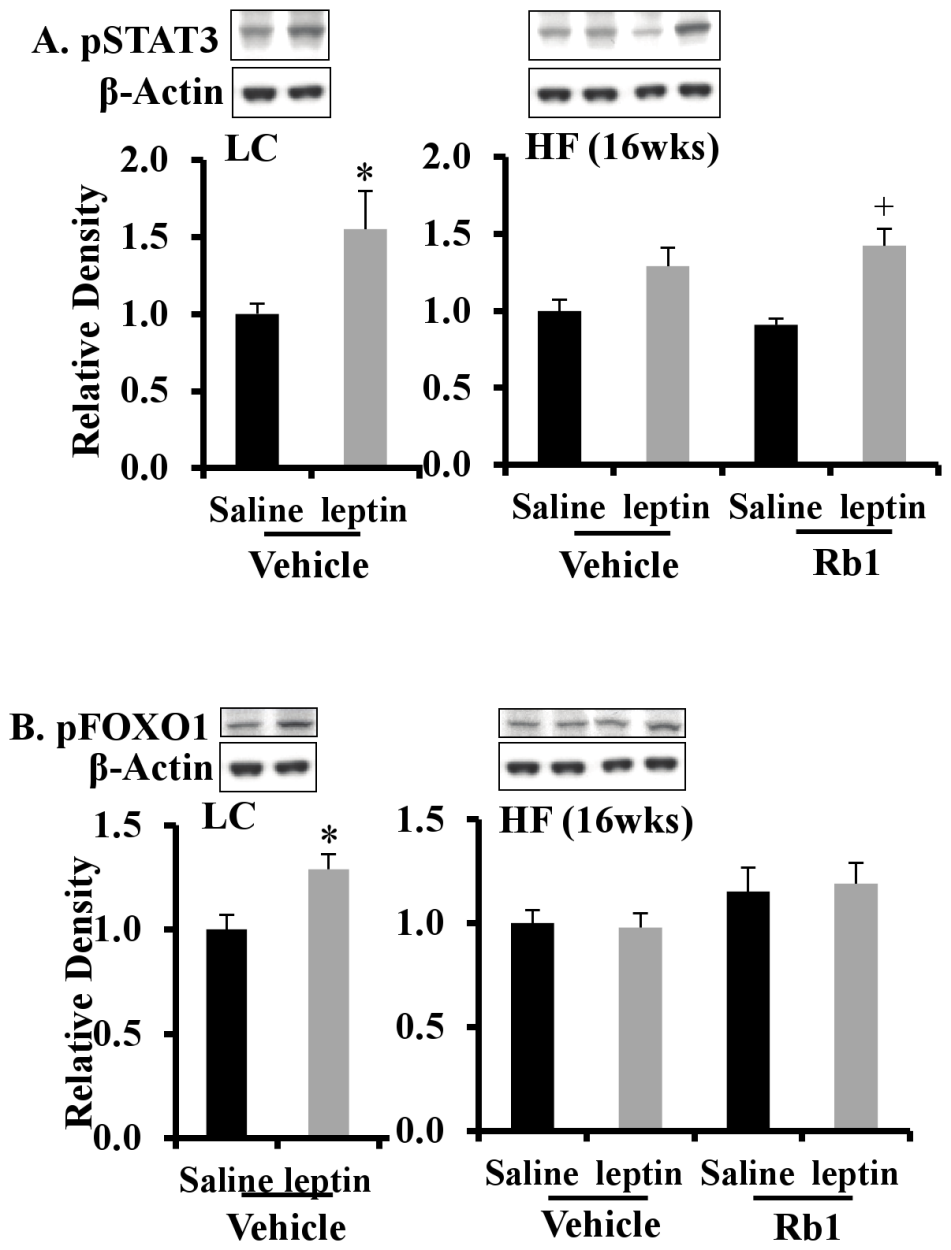
Chronic Rb1 treatment improved central leptin signaling in obese mice fed a HF diet for 16 weeks. The phosphorylation of STAT3 (A) and FOXO1 (B) in the mediobasal hypothalamus 1 hour after the icv injection of leptin or saline in mice fed a LC diet and obese mice fed a HF diet for 16 weeks with or without chronic treatment of Rb1 (n = 6–8). *p<0.05 vs. (vehicle + saline) group; ^#^ *p*<0.05 vs. (vehicle + Leptin) group; +p<0.05 vs. (Rb1 + saline) group. Data are presented as mean ± SEM.

To clarify the mechanism by which chronic Rb1 treatment improved leptin sensitivity, protein expression of the leptin signaling molecules p-STAT3 and p-FOXO1 was measured in the mediobasal hypothalamus. Icv injection of leptin increased p-STAT3 (+55%, *p*<0.05) in mice fed LC diet, while these responses were not observed in HF diet-induced obese mice (Fig. 6A). After Rb1 treatment, the response of leptin signaling molecules was restored, with a 42% increase in p-STAT3 (*p*<0.001) following leptin administration to Rb1-treated HF mice (Fig. 6A). Leptin also increased phosphorylation of FOXO1 in the mediobasal hypothalamus of LC diet fed mice, a response that was blunted in HF mice (Fig. 6B). However, in this case Rb1 treatment did not restore the leptin-induced increase in p-FOXO1 (Fig. 6B). Therefore, in the mediobasal hypothalamus, Rb1 acted on the STAT3 pathways rather than the FOXO1 pathway to restore leptin signaling.

### Rb1 treatment affected the hypothalamic neuropeptides regulating energy balance

The effect of Rb1 treatment on hypothalamic neuropeptides expression was examined to investigate the mechanisms by which this compound suppressed food intake and body weight gain. Rb1 treatment significantly increased anorexigenic pro-opiomelanocortin (POMC, +75%; p<0.05) and decreased orexigenic agouti-related protein (AgRP, -24%; *p*<0.05) mRNA expression in the mediobasal hypothalamus of HF mice, but had no effect on orexigenic neuropeptide Y (NPY) mRNA levels (Table 2).

**Table 2:** Relative mRNA expression of neuropeptides in the mediobasal hypothalamus in obese mice fed a HF diet for 16 weeks with or without Rb1 chronic treatment.

	HF	Rb1	*p*-value
POMC	1.00±0.14	1.75±0.23^*^	0.034
AgRP	1.00±0.05	0.76±0.07^*^	0.045
NPY	1.00±0.04	0.90±0.09	0.464

HF: high-fat diet-induced obese mice; Rb1: high-fat diet-induced obese mice treated with ginsenoside Rb1. *p<0.05 vs. HF group. Data are presented as Mean ± SEM.

## Discussion

In the current study, Rb1 prevented body weight gain and reduced fat mass in obese mice fed a HF diet. Rb1 also decreased average food intake during the course of this study. This is similar to the study by Xiong and colleagues, which showed that Rb1 has an anti-obesity effect in rats and its suppression of food intake is not due to malaise, as attested by a conditioned taste aversion test [21]. Importantly, our study extends the mechanism of Rb1 in suppressing food intake. Rb1 treatment increased the anorexigenic hormone, peptide YY (PYY), in the blood and modulated hypothalamic neuropeptides, specifically by increasing anorexigenic POMC and decreasing orexigenic AgRP mRNA expression in HF diet-induced obese mice. Rodent models of HF diet-induced obesity are characterized by inflammation in both peripheral tissues and in the hypothalamic regions critical for energy homeostasis [4], [8], which is considered an important mechanism linking obesity to glucose intolerance, insulin resistance and leptin resistance. This study has demonstrated that ginsenoside Rb1 treatment provides an anti-obesity-associated inflammatory effect, with the pronounced reduction of peripheral and hypothalamic inflammation and improvement of glucose tolerance and central leptin resistance in HF diet-induced obese mice.

Overnutrition and obesity induce inflammatory responses in peripheral metabolic tissues, which decreases insulin sensitivity in target cells (adipocytes and hepatocytes) and contributes to glucose intolerance and the development of type 2 diabetes [2], [8], [9], [29]. For example, in obese rodents fed a HF diet macrophages infiltrate the liver and increase the mRNA expression of the pro-inflammatory cytokines TNF-α, IL-1β and IL-6 [2], [9]. These cytokines activate NF-κB signaling in hepatocytes, causing hepatic insulin resistance and glucose intolerance [2], [3]. In the current study, Rb1 decreased the level of pro-inflammatory cytokines (TNF-α, IL-1β and IL-6) and the inflammatory signaling molecule (p-IKK) in the adipose tissue and liver, which may have contributed to the improved glucose tolerance observed in Rb1 treated diet-induced obese mice. Furthermore, it is well-documented that adiponectin ameliorates insulin resistance and reduces fatty acid levels in rodents [30], by decreasing hepatic gluconeogenesis and increasing lipid oxidation in muscle [31], [32]. In this study, the increased adiponectin levels after Rb1 treatment may contribute to the improved glucose tolerance and reduced fat accumulation that we observed in obese mice treated with this compound.

Our study demonstrated that chronic treatment with Rb1 suppressed inflammation in the mediobasal hypothalamus of diet-induced obese mice, as shown by decreased protein expression of IL-6, IL-1β and p-IKK in this region. In accordance with our results, another study demonstrated that acute oral administration of Rb1 significantly reduced IL-6, IL-1β and TNF-α mRNA expression in mouse brain tissue, and inhibited morphological activation of microglia following intraperitoneal injections of lipopolysaccharide endotoxin [33]. Recently, Thaler and colleagues demonstrated that mice and rats fed a HF diet had increased TNF-α and IKK/NFκB mRNA expression in the hypothalamus [4]. Hypothalamic inflammation is considered a key pathology of obesity in rodents and humans [34], leading to central leptin resistance through activation of the negative regulators of leptin signaling, SOCS and PTP1B [5], [12]. Our results demonstrate that Rb1 decreased the upregulation of SOCS3 and PTP1B in the hypothalamus of HF diet-induced obese mice. Therefore, the inhibition of SOCS3 and PTP1B and attenuation of hypothalamic inflammation, contributes to the therapeutic effect of Rb1 on central leptin resistance observed in our mouse model.

The adipocyte-derived hormone leptin promotes negative energy balance through various signaling pathways (STAT3 and FOXO1) in the hypothalamus. Constitutive activation of the inflammatory signaling molecule IKKβ in the hypothalamus of mice impaired STAT3 phosphorylation in response to central leptin administration and induced central leptin resistance [5]. In our study, leptin induced phosphorylation of STAT3 was restored after Rb1 treatment in HF mice, suggesting the anti-inflammatory properties of Rb1 (inhibition of p-IKK in the hypothalamus) may contribute to this effect. FOXO1, a member of forkhead box containing protein O superfamily, is highly expressed in the hypothalamus and contributes to anorexigenic effect of leptin [35], [36]. Leptin signaling through the PI3K/Akt pathway induces FOXO1 phosphorylation and degradation, decreasing FOXO1 activity in the hypothalamus [35], [36]. Phosphorylation of FOXO1 results in its export from the nucleus and allows p-STAT3 to bind to neuropeptide promoters, stimulating transcription of anorexigenic pro-opiomelanocortin (POMC) and inhibiting orexigenic agouti-related protein (AgRP) expression [37]. In our study, central leptin injections stimulated FOXO1 phosphorylation in LC mice, but not in HF mice with central leptin resistance. The inhibition of leptin-induced FOXO1 phosphorylation in obese mice with central leptin resistance indicates the impairment of leptin signaling occurs at the step of FOXO1 phosphorylation or upstream. In the current study treatment of obese mice with Rb1 restored leptin induced activation of STAT3 phosphorylation, but not phosphorylation of FOXO1 in the mediobasal hypothalamus. Therefore, in the hypothalamus, Rb1 acted on the STAT3 signaling pathway rather than the FOXO1 pathway to restore leptin signaling and sensitivity, although the precise mechanisms need further examination.

The anti-inflammatory and leptin sensitizing effects of Rb1 treatment could be due to multiple factors. It is likely that reduced food intake leads to a reduction in fat deposition that in turn reduces pro-inflammatory cytokines, glucose intolerance and improves leptin sensitivity. However, a direct effect of Rb1 of inhibiting inflammation cannot be completely excluded [18], [21]. For example, it has been reported in an in vitro study that Rb1 directly inhibited inflammatory responses in rat aortic smooth muscle cells [18]. Furthermore, in a rat study by Xiong et al, pair-fed rats with comparable food intake to Rb1 treatment rats did not show the improved glucose tolerance evident in Rb1 treated animals, suggesting a direct effect of Rb1 in improving glucose metabolism [21].

Peptide YY (PYY) is a gut-brain anorexigenic hormone that promotes negative energy balance by reducing appetite [38]. Peripheral infusion of PYY reduces food intake in rodents [39], and transgenic mice overexpressing PYY have increased plasma PYY concentrations, and are protected against diet-induced obesity [38]. In the present study, the increased plasma PYY in Rb1 treated HF diet-induced obese mice may have contributed to the negative energy balance, lower body weight gain and fat accumulation in these animals. The mechanism by which Rb1 treatment increased circulating PYY levels remains to be determined. However, PYY is predominantly secreted by intestinal L cells located in the distal gastrointestinal tract [40], and it has been reported that the PYY levels are decreased in patients with inflammatory bowel disease [41]. In addition, three days of oral Rb1 treatment potently inhibited the expression of TNF-α and IL-1β in the inflamed colon of mice with colitis [19]. Since the colon of obese mice overexpresses pro-inflammatory cytokines [42], an anti-inflammatory effect of Rb1 in the gastrointestinal tract may have increased PYY secretion in obese mice.

The melanocortin system comprises anorexigenic POMC expressing neurons and orexigenic AgRP expressing neurons in the arcuate nucleus of the mediobasal hypothalamus [43]. α-melanocortin-stimulating hormone (α-MSH), a post-translational product of the POMC gene, binds to the melanocortin receptor 4 (MC4R) and triggers an anorectic signal in the hypothalamus, while AgRP (an inverse agonist of MC4R) prevents α-MSH binding to MC4R. Chronic Rb1 treatment significantly increased POMC and inhibited AgRP mRNA expression in high-fat diet fed mice, implying that Rb1 exerts its anorexigenic action at least partially by targeting the melanocortin system, the POMC and AgRP neurons. It is known that these neurons located in the mediobasal hypothalamus receive and integrate the signaling of various gut and adipostatic hormones, including PYY, leptin and insulin. In our study, the effect of Rb1 treatment on the hypothalamic melanocortin system may be due to increased plasma PYY, and the improvement of hyperleptinemia and central leptin sensitivity after Rb1 treatment.

In summary, this study has demonstrated that ginsenoside Rb1 treatment inhibits inflammation in the adipose tissue, liver and hypothalamus of HF diet induced obese mice. Treatment with Rb1 resulted in the improvement of glucose tolerance, central leptin sensitivity and hypothalamic leptin signaling (p-STAT3). We have also shown that Rb1 treatment increased the circulating concentrations of the anorexigenic hormone PYY and regulated melanocortin POMC/AgRP neuropeptides in the mediobasal hypothalamus, which contribute to negative energy balance. Ginsenoside Rb1 has the potential for use as an antiobesity therapeutic agent that functions by modulating obesity-induced inflammation and improving central leptin sensitivity in HF diet-induced obesity.

## Supporting Information

Table S1 **The primers used in qPCR for neuropeptide mRNA measurement.**
(DOCX)

Table S2 **Effects of acute Rb1 administration (14 mg/kg, ip, two days) on body weight and food intake in obese mice after a high-fat diet feeding for 8 weeks.**
(DOCX)
