# Supplementary material for: Central Inflammation and Leptin Resistance Are Attenuated by Ginsenoside Rb1 Treatment in Obese Mice Fed a High-Fat Diet
Source: PLoS One. 2014 Mar 27;9(3):e92618. doi: 10.1371/journal.pone.0092618 (PMC3968027; doi:10.1371/journal.pone.0092618)
Supplement: Table S1 — The primers used in qPCR for neuropeptide mRNA measurement. (DOCX) [file pone.0092618.s001.docx]

Table S1 The primers used in qPCR for neuropeptide mRNA measurement

| GENE | Forward primer | Reverse primer | NCBI reference |
| --- | --- | --- | --- |
| NPY | ATACTACTCCGCTCTGCGAC | GTGTCTCAGGGCTGGATCT | NM_023456.2 |
| AgRP | AGTTGTGTTCTGCTGTTGGC | CTGATGCCCTTCAGTGGAG | NM_007427.2 |
| POMC | CCATAGATGTGTGGAGCTGG | CCAGCGAGAGGTCGAGTT | NM_008895.3 |
| γ-actin | GCTAACAGAGAGAAGATGACG | CAGATGCATACAAGGACAGC | NM_009609.2 |
