# Supplementary material for: Central Inflammation and Leptin Resistance Are Attenuated by Ginsenoside Rb1 Treatment in Obese Mice Fed a High-Fat Diet
Source: PLoS One. 2014 Mar 27;9(3):e92618. doi: 10.1371/journal.pone.0092618 (PMC3968027; doi:10.1371/journal.pone.0092618)
Supplement: Table S2 — Effects of acute Rb1 administration (14 mg/kg, ip, two days) on body weight and food intake in obese mice after a high-fat diet feeding for 8 weeks. (DOCX) [file pone.0092618.s002.docx]

Table S2 Effects of acute Rb1 administration (14 mg/kg, ip, two days) on body weight and food intake in obese mice after a high-fat diet feeding for 8 weeks

|  | HF |  | HF + Rb1 |
| --- | --- | --- | --- |
| BW before Rb1 treatment (g) | 29.84 ± 0.49 |  | 29.59 ± 0.47 |
| BW after Rb1 treatment (g) | 30.12 ± 0.47 |  | 29.89 ± 0.43 |
|  |  |  |  |
| FI of 24 hours after Rb1 treatment (g) | 3.39 ± 0.16 |  | 3.44 ± 0.10 |

HF: high-fat diet-induced obese mice; Rb1: ginsenoside Rb1 treatment; BW: body weight; FI: food intake. Data are presented as mean ± SEM.
